# The universality of eAREs in animal feces suggesting that eAREs function possibly in horizontal gene transfer

**DOI:** 10.5455/javar.2023.j658

**Published:** 2023-03-31

**Authors:** Yusha Jiang, Lang Zhao, Jia Danyang Li, Jialiang Sun, Rui Miao, Bo Shao, Peifu Wu

**Affiliations:** College of Life Sciences, Southwest Forestry University, Kunming, China

**Keywords:** eAREs, iAREs, PCR, animal feces, guts, horizontal gene transfer

## Abstract

**Objectives:**

This study aimed to pinpoint the universality of extracellular antimicrobial resistance elements (eAREs) and compare the contents of eAREs with those of intracellular AREs (iAREs) in animal feces, thus laying a foundation for the further analysis of the horizontal transfer of antimicrobial resistance genes (ARGs) in the animal guts.

**Materials and Methods:**

Extracellular DNAs were isolated from the fecal samples of *Pavo cristatus* (*n* = 18), *Ursus thibetanus* (*n* = 2), two breeds of broilers (*n* = 21 and 11, respectively), and from the contents of rabbit intestines (*n* = 5). eAREs were detected by PCR technology. iAREs in *P. cristatus* and broiler feces were also detected and compared with the corresponding eAREs. In addition, some gene cassettes of class 1 integrons were sequenced and analyzed.

**Results:**

The results showed that eAREs exist in animal feces and intestinal contents. In this study, different eAREs were detected from animal feces and intestinal contents, and *tetA*, *tetB*, *sul1*, *sul2*, class 1 integron, and IncFIB presented the highest detection rates. The detection rates of certain eAREs were significantly higher than those of parallel iAREs. The integral cassettes with intact structures were found in eAREs, and the cassettes carried ARGs.

**Conclusions:**

The presented study here sheds light on the presence of eAREs in animal feces or guts, and eAREs may play an important role in the horizontal gene transfer of ARGs.

## Introduction

The emergence of multidrug-resistant bacteria or “superbugs” has brought unprecedented challenges to clinical treatment, such as reduced categories of available antibiotics, low efficiency of clinical treatment, and increases in treatment cost and mortality [1,2]. The evidence has continuously shown that antimicrobial resistance has displayed its existence in various environments, such as human or animal hospitals [3–5], indigenous residents, wild animals [6], and water circumstances (including rivers, lakes, oceans, etc.) [7,8], soil [9], polar lands [10], food, sewage, sludge [11,12], and air [13]. While antimicrobial resistance genes (ARGs) have shared their specific ways to transfer via the horizontal genetic elements (HGEs), such as plasmids, integrons, transposons, and bacteriophages, among or between different species [14–18]. Data analyses of structural characteristics of bacterial and microbiota metagenomes again provided convincible proof of the patterns of various levels of gene transfer resulting in a great influence on the acquirable genotypes of pathogenic species or microbiota [19–21]. Genes encoding integrases, transposases, mobile ARGs, and their genetic backgrounds, have shown clues of gene transfers within the same species or between closely related bacteria [19–21].

There exist two forms of ARGs, intracellular ARGs (iARGs) and extracellular ARGs (eARGs). Horizontal gene transfer (HGT) of iARGs was observed, as showed in plenty of literature, either through the binding pathway of intercellular contact or through the transduction mediated by phage infection [22,23]. eARGs persist in the form of extracellular DNAs (eDNAs). They could be absorbed into competent cells showing no resistance phenotypes in the environment via natural transformation, thus leading to the emergence and dissemination of drug resistance [24–27]. It has been demonstrated that extracellular DNA, which is widely and persistently distributed in environmental samples such as soil, sediment, and feces, accounts for a large percentage of total DNA [28]. It can be concluded that eDNAs in the microbial niche act as a persistent and dynamic gene pool, contributing to the natural transformation of competent cells.

Until now, best to our knowledge, the studies on ARG transfer in humans and animals mainly focused on iARGs rather than eARGs. Although eDNAs in the environment contain a large number of eARGs, even mobile elements, and have functioned in the HGT of ARGs [29,30], there are still no associated reports on eDNAs in human and animal microbiota. In this study, followed by the purification of the eDNAs from the feces of *Pavo cristatus*, *Ursus thibetanus*, two breeds of broilers along with the contents of rabbit intestines, ARGs, integrons, integral gene cassettes, and plasmid replicons were detected from eDNAs respectively to analyze the potential universality and role of extracellular antimicrobial resistance elements (eAREs) in the animal intestine during the horizontal transfer of resistance elements. In addition, intracellular antimicrobial resistance elements (iAREs) in the isolated iDNAs from samples of *P. cristatus* and broiler feces were also detected, then the divergences between eAREs and iAREs were compared. Some class 1 integral cassettes were sequenced and analyzed to imply the possibility of eAREs being involved in the horizontal transfer of drug resistance.

## Materials and Methods

### Sample collection

A total of 52 fecal samples were collected in this study, including 18 from *P. cristatus*, 21 from Wuding broilers, 11 from Sanhuang broilers, and 2 from *U. thibetanus*. The collected fecal samples were kept in the 0°C incubator and immediately sent to the laboratory for further testing. In addition, in order to confirm whether eDNAs exist in the gut contents of the animals, the contents of ceca, colons, and rectums from five rabbits (*Oryctolagus cuniculus*) were also collected respectively.

### Purification of eDNAs and iDNAs

The eDNAs were isolated from collected feces and intestinal contents, respectively. Briefly, the 2 gm sample was washed twice with sterile PBS. Then 0.2 gm polyvinylpolypyrrolidone and 4 ml NaH_2_PO_4_ solution (0.12 M) were added, mixed well, and incubated for 1 h at 25°C with a rotation 250 rpm. The suspension was centrifuged at 12,000 rpm, and the supernatant was obtained and filtered with a 0.22 μm membrane. Then, after adding 10 μl protease K (20 mg/ml, BioTeke Corporation, China) into the supernatant, the solution was incubated at 55°C for 15 min. 1% CTAB solution (pH 8.0), and NaCl (with a final concentration of 2 M) were then added. The mixture was again incubated at 65°C for 10 min. Finally, the eDNAs were precipitated by adding sodium acetate solution (0.12 M) and cooling absolute ethanol.

The iDNAs were isolated from collected feces of *P. cristatus* and two breeds of broilers, following the instructions of a stool genomic DNA extraction kit (QIAGEN, Germany).

### PCR detection of eAREs and iAREs

Three classes of integrase genes (class 1, 2, and 3 integrons), 13 ARGs, and 10 types of plasmid replicons were detected (the primers used in this study and targeted genes information were shown in Table 1). The ARGs included *bla*_SHV_, *bla*_TEM_, *bla*_CTX-M1_, *bla*_CTX-M2_, *bla*_CTX-M9_, *bla*_CTX-M8/25_, *bla*_NDM_, *CLR5*, *tetA*, *tetB*, *sul1*, *sul2,* and *sulA*. The 10 replicons were IncB/O, IncFIC, IncP, IncFIIA, IncFIA, IncFIB, IncI1, IncFrep, IncN, and IncL/M. A total of 25 μl amplification reaction system was adopted to detect the above genes, containing 1 μl of each of the oligonucleotide primers (0.4 mM), 12.5 μl 2 × Taq Master Mix (BioTeke Corporation, China), 1 μl DNA templates, and 9.5 μl ddH_2_O. The amplification was performed in a thermal cycler with amplification conditions as follows: denaturation for 5 min at 94°C; 30 cycle program as a denaturation for 45 sec at 94°C, primer annealing for 30 sec at the corresponding temperature listed in Table 1; extension for 50 sec at 72°C; and finally total extension for 6 min at 72°C. The PCR products were checked by electrophoresis through 1% agarose gel.

### Amplification and sequencing of the class 1 integral cassettes from eDNAs

The class 1 integral cassettes were amplified using eDNAs isolated in this study. The primers are shown in Table 1, and the amplification scheme is described above. After the electrophoresis of the PCR products, bands of different sizes were observed. Here, the products with high-density bands between 1,000 and 1,500 bp were selected randomly and sequenced.

### Analysis of sequencing data

Based on the NCBI RefSeq database, the sequenced data in this study were aligned with the annotation files through the blastn program to analyze the structures of integral cassettes. The NCBI and ResFinder databases were used to compare and analyze the resistance genes in the cassettes.

### Statistical analysis

IBM SPSS Statistics 22 was used for statistical analysis. A *p*-value of ≤0.05 was considered significant.

**Table 1.** The primers and information of targeted genes used in this study.

Targeted genes	Primer sequence (5´-3´)	Product size (bp)	Annealing temperature (°C)
Integrase 1	F: ACGAGCGCAAGGTTTCGGT	565	52
	R: GAAAGGTCTGGTCATACATG		
Integrase 2	F: GTGCAACGCATTTTGCAGG	403	52
	R: CAACGGAGTCATGCAGATG		
Integrase 3	F: CATTTGTGTTGTGGACGGC	717	52
	R: GACAGATACGTGTTTGGCAA		
Integral cassettes	F: GGCATACAAGCAGCAAGC	Variable	45
	R: AAGCAGACTTGACCTGAT		
*bla*_SHV_	F: AGCCGCTTGAGCAAATTAAAC	786	55
	R: GTTGCCAGTGCTCGATCAGC		
*bla*_TEM_	F: CATTTCCGTGTCGCCCTTATTC	846	55
	R: CCAATGCTTAATCAGTGAGGC		
*bla*_CTX-M1_	F: CGTCACGCTGTTGTTAGGAA	781	55
	R: ACGGCTTTCTGCCTTAGGTT		
*bla*_CTX-M2_	F: CTCAGAGCATTCGCCGCTCA	843	55
	R: CCGCCGCAGCCAGAATATCC		
*bla*_CTX-M9_	F: GCGCATGGTGACAAAGAGAGTGCAA	876	55
	R: GTTACAGCCCTTCGGCGATGATTC		
*bla*_CTX-M8/25_	F: CCAGGCGAACGATGTTCAACA	730	55
	R: CGGCTCCGACTGGGTGAAGTA		
*bla*_NDM_	F: GGTTTGGCGATCTGGTTTTC	621	55
	R: CGGAATGGCTCATCACGATC		
*CLR*5	F: CGGTCAGTCCGTTTGTTC	309	55
	R: CTTGGTCGGTCTGTAGGG		
*tet*A	F: GCTACATCCTGCTTGCCTTC	210	54.6
	R: CATAGATCGCCGTGAAGAGG		
*tet*B	F: GGTTGAGACGCAATCGAATT	206	52.9
	R: AGGCTTGGAATACTGAGTGTAA		
*sul*1	F: CGCACCGGAAACATCGCTGCAC	163	55.8
	R: TGAAGTTCCGCCGCAAGGCTCG		
*sul*2	F: GCACTCCAGCAGGCTCGTAA	191	60
	R: CGGGAATGCCATCTGCCTTGAG		
*sul*A	F: GCACTCCAGCAGGCTCGTAA	198	56.8
	R: CTCTGCCACCTGACTTTTCCA		
IncB/O	F: GCGGTCCGGAAAGCCAGAAAAC	159	60
	R: TCTGCGTTCCGCCAAGTTCGA		
IncFIC	F: GTGAACTGGCAGATGAGGAAGG	262	60
	R: TTCTCCTCGTCGCCAAACTAGAT		
IncP	F: CTATGGCCCTGCAAACGCGCCAGAAA	534	60
	R: TCACGCGCCAGGGCGCAGCC		
IncFIIA	F: CTGTCGTAAGCTGATGGC	270	60
	R: CTCTGCCACAAACTTCAGC		
IncFIA	F: CCATGCTGGTTCTAGAGAAGGTG	462	60
	R: GTATATCCTTACTGGCTTCCGCAG		
IncFIB	F: GGAGTTCTGACACACGATTTTCTG	702	60
	R: CTCCCGTCGCTTCAGGGCATT		
IncI1	F: CGAAAGCCGGACGGCAGAA	139	60
	R: TCGTCGTTCCGCCAAGTTCGT		
IncFrep	F: TGATCGTTTAAGGAATTTTG	270	60
	R: GAAGATCAGTCACACCATCC		
IncN	F: GTCTAACGAGCTTACCGAAG	559	60
	R: GTTTCAACTCTGCCAAGTTC		
IncL/M	F: GGATGAAAACTATCAGCATCTGAAG	785	60
	R: CTGCAGGGGCGATTCTTTAGG		

## Results

### Detection of integrons from iDNAs and eDNAs

Integron 3 was not detected in any samples, and no integrons of any type were detected in the feces of *U. thibetanus*.

**Figure 1.**
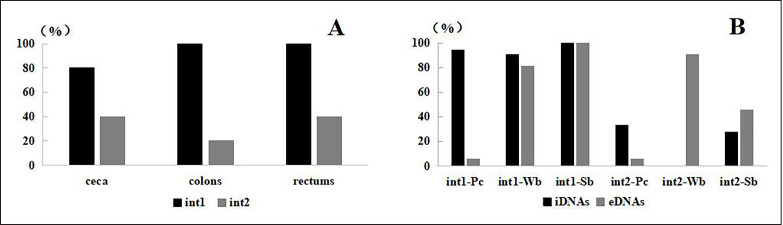
Detection rates of different integrons from rabbit intestinal contents (A) and broilers (B). In the figure, Pc is *P. cristatus*, Wb is Wuding broilers, Sb is Sanhuang broilers, int1 is integron 1, and int2 is integron 2.

Class 1 integron amplified using iDNAs (iInt1) or eDNAs (eInt1) was found in all samples (Fig. 1). For fecal samples of *P. cristatus*, the detection rate of iInt1 (94.44%) was significantly higher than that of eInt1 (5.56%) (*p *< 0.01). There was no difference between the detection rate of iInt1 and that of eInt1 in both broiler samples, and no noticeable difference in iInt1 detection rates was observed between peacock and broiler samples. In contrast, the rate (5.56%) of eInt1 in peacock samples was significantly lower than that in broiler samples (*p *< 0.01). The detection rate of eInt1 in rabbit ceca was 80%, while both were 100% in colons and rectums.

Class 2 integron amplified using iDNAs (iInt2) or eDNAs (eInt2) was detected in all samples except iInt2 in samples of Wuding broilers (Fig. 1). For the feces of *P. cristatus*, the detection rate of iInt2 (33.33%) was significantly higher than that of eInt2 (5.56%) (*p *< 0.05). Interestingly, in samples of Wuding broilers, although iInt2 was not detected, the rate of eInt2 was very high (90.48%). For samples of Sanhuang broilers, the rate of eInt2 (45.45%) was also higher than that of iInt2 (27.27%) (*p *< 0.05). The detection rate of eInt2 in samples of Wuding broilers (90.48%) was significantly higher than in samples of *P. cristatus* (5.56%) and Sanhuang broilers (45.45%) (*p *< 0.01). The detection rates of eInt2 in rabbits’ cecum and rectum were 40%, while the rate in the colon was 20%.

### Detection of ARGs from iDNAs and eDNAs

The ARGs amplified using iDNAs were written as “i + abbreviation name of corresponding ARGs” for convenience. The ARGs derived from eDNAs were written as “e + abbreviation name of corresponding ARGs.”

**Figure 2.**
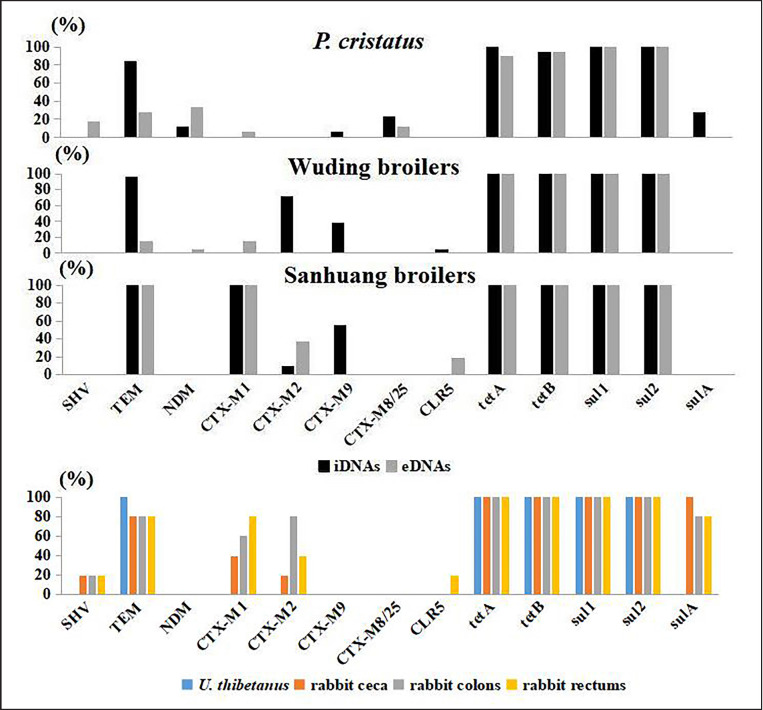
Detection rates of different ARGs.

Overall, the detection rates of genes *tetA*, *tetB*, *sul1*, and *sul2* were very high. The rates of i*tetA*, i*tetB*, i*sul1*, i*sul2*, e*tetA*, e*tetB*, e*sul1*, and e*sul2 *were all 100% except e*tetA*, i*tetB*, and e*tetB *in peacock feces (Fig. 2). The detection rate of *bla*TEM was the second. The detection rates of i*bla*TEM in the feces of peacocks and Wuding broilers (83.33% and 95.24%, respectively) were significantly higher than those of e*bla*TEM (27.78% and 14.29%, respectively) (*p *< 0.01). The detection rates of i*bla*TEM and e*bla*TEM in Sanhuang broilers and e*bla*TEM in *U. thibetanus* were all 100%. The rates of e*bla*TEM in rabbit intestinal contents were all 100%. The detection rates of *bla*CTX-M1 were also relatively high, especially in the feces of Sanhuang broilers, which presented 100% detection rates of both i*bla*CTX-M1 and e*bla*CTX-M1.

Interestingly, the detection rates of several eARGs were significantly higher than iARGs (Fig. 2). Although i*bla*SHV and i*bla*CTX-M1 were not detected in peacock feces, the detection rates of e*bla*SHV, e*NDM*, and e*bla*CTX-M1 were significantly higher than those of the corresponding iARGs (*p *< 0.05). Although no i*NDM* or i*bla*CTX-M1 were found, e*NDM* and e*bla*CTX-M1 were detected in the feces of Wuding broilers. e*CLR5*, but i*CLR5 *was observed, and the detection rate of e*bla*CTX-M2 was significantly higher than that of i*bla*CTX-M2 in Sanhuang broiler feces (*p *< 0.05).

The types and rates of ARGs carried by different animals varied. *bla*CTX-M8/25 was only detected in the eDNAs and iDNAs of peacock feces. i*NDM* was found only in peacock feces, while e*NDM* was observed in peacock and Wuding broiler feces.

### Detection of plasmid replicons from iDNAs and eDNAs

For convenience, the plasmid replicons amplified from iDNAs were written as “i + abbreviation name of corresponding replicons.” In contrast, the replicons derived from eDNAs were written as “e + abbreviation name of corresponding replicons.”

iIncFIB and eIncFIB were detected in all samples (Table 2), and IncFIB was also the replicon with the highest average detection rate. The detection rates of iIncFIB were significantly higher than those of eIncFIB in the feces of peacocks and Wuding broilers (*p *< 0.01). IncFIIA was not detected in any samples. IncL/M was only found in the colon contents of rabbits, while IncB/O was only amplified using eDNAs from the feces of Wuding broilers.

Again, interestingly, the detection rates of several plasmid replicons using eDNAs were higher than iDNAs (Table 2). No iIncP was detected, but higher eIncP was observed in peacock feces (*p *< 0.05). No iIncB/O was detected, but the rate of eIncB/O was 14.29% in Wuding broiler feces. The detection rates of eIncFIC and eIncP were slightly higher than those of iIncFIC and iIncP, respectively. In contrast, the detection rate of eIncFrep was significantly higher than that of iIncFrep in Wuding broiler feces (*p *< 0.01). The detection rates of eIncFIA and eIncN were also higher than those of iIncFIA and iIncN in Sanhuang broiler feces (*p *< 0.05).

### Examples of the structures of class 1 integral cassettes in eDNAs

In this study, the gene cassettes of class 1 integrons were amplified using the eDNAs, and three PCR products were selected respectively from Wuding broiler feces and rabbit intestinal contents for further sequencing. The sequencing sizes from Wuding broiler feces were all 990 bp, and the result sequences all contained the *aadA1* gene expressing streptomycin 3’’ - O-adenyltransfer.

**Figure 3.**
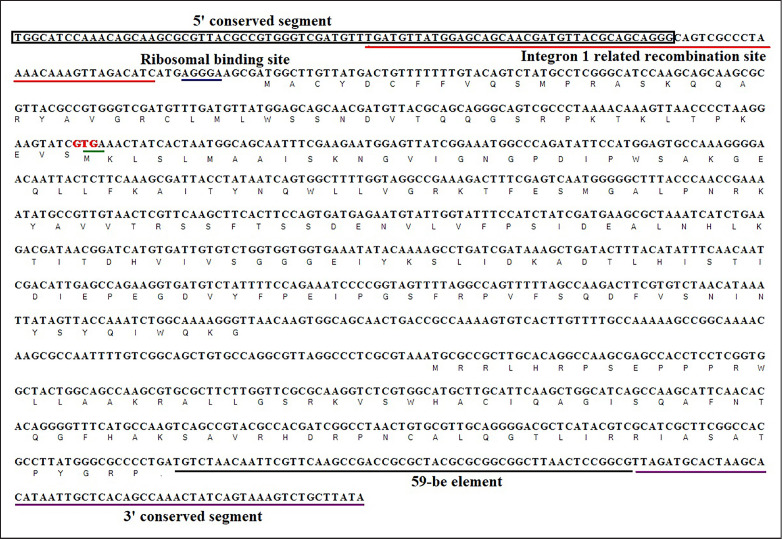
Structural analysis of integron 1 gene cassette. The rectangular box displayed 5’ conserved segment of class 1 integron; The red underline showed the integron 1 related recombination site; The blue underline showed the ribosome binding site; The green underline indicated the stop codon of the first hypothetical protein gene; The red letters presented the start codon of gene *dfra15*; The coding frame followed the gene *dfra15 *was the second hypothetical protein gene; Black underline showed 59-be element; The purple underline showed class 1 integron 3’ conserved segment.

The sequencing sizes of amplified products from rabbit samples were all 1,212 bp. The cassette possessed an intact structure, including 5’ conserved segment, integron 1 related recombination site, ribosomal binding site, hypothetical protein gene, *dfra15* resistance gene conferring resistance to sulfonamide, orf126 hypothetical protein gene, 59-be element, and 3’ conserved segment of class 1 integron (Fig. 3). The open reading frame of the first putative protein overlapped with the coding frame of the *dfra15* gene by 4 bases (the NCBI accession number is ON243888).

## Discussion

It has been shown that antibiotic-resistant elements have occupied almost every dwelling niche of microbes [31,32]. Antimicrobial resistance observed in the clinic is closely related to the selection pressure caused by the overuse or irrational use of antibiotics. Thus, the use of antibiotics has directly contributed to the emergence and horizontal transfer of resistance genes. Silva et al. [33] analyzed the resistance patterns of *Escherichia coli* and *Enterococcus* isolated from the intestinal contents of wild rabbits. The authors observed resistance genes *bla*TEM, *aadA*, *aac(3)*-II, *tetA*, *tetB*, *catA*, *sul1*, *sul2*, and *sul*3 in *E. coli* isolates and found *aac(6’)-aph(2’’)*, *ant(6)*-Ia, *tetM*, *tetL*, *aph(3’)*-IIIa, *ermB*, and *vatD* in *Enterococcus*. Chen et al. [34] witnessed 194 resistance genes, including genes highly similar to those observed in clinical isolates, from terrestrial and aquatic vertebrates using the method of transcriptional analysis. They found that the genes associated with clinical resistance were always co-located with mobile elements, indicating the intrinsic feature of gene transmission through HGT. Forsberg et al. [35] confirmed a recent ARG exchange between environmental microbiota and clinical pathogenic isolates. 

Multidrug-resistant soil microorganisms carried five classes of gene cassettes (β-lactams, aminoglycosides, amphenicols, sulfonamides, and tetracyclines). The high similarity of the nucleotide sequences between the gene cassettes analyzed in this study and those corresponding to human pathogens confirms the general existence of gene exchanges. Many studies have proven the universality and transferability of resistance genes among people, animals, and the environment. More evidence is still needed, especially in some special cases or in general conditions, to explore the preference of climate, region, species, and niche shown by the universality and transferability of ARGs.

**Table 2.** The detection rates of plasmid replicons (%).

Host	location	B/O	FIC	P	FIA	FIB	I1	Frep	N	L/M
*P. cristatus*	iDNAs	0	0	0	33.33	61.11	0	0	11.11	0
	eDNAs	0	0	50	16.67	5.56	0	0	0	0
Wuding broilers	iDNAs	0	4.76	4.76	0	66.67	28.57	4.76	0	0
	eDNAs	14.29	9.52	9.52	0	9.52	4.76	61.90	0	0
Sanhuang broilers	iDNAs	0	54.55	27.27	0	100	0	45.45	18.18	0
	eDNAs	0	45.45	27.27	9.09	90.91	0	18.18	36.36	0
*U. thibetanus*	eDNAs	0	0	0	0	100	100	100	0	0
Rabbit ceca	eDNAs	0	80	20	0	20	0	0	0	0
Rabbit colons	eDNAs	0	20	60	0	40	0	20	20	20
Rabbit rectums	eDNAs	0	100	60	0	60	0	0	0	0

HGT is an important mechanism of resistance gene transmission among different species or genera [19–23]. Through HGT, bacteria could obtain important adaptive phenotypes, such as virulence, beneficial metabolic pathways, and antibiotic resistance. Till now, the research on the antimicrobial resistance of human-and-animal-associated bacteria, or microbiota, was mainly confined to iDNAs at the molecular level, and though it has achieved great achievements, no documents on eDNAs have been recorded so far. *In silico* analysis of environmental metagenomic data on soil, water, and sludge [36], etc., has demonstrated that the environment inhabited by microbiota is a vast reservoir of resistance genes, including the abundance of mobile ARGs found today. In addition, the confirmed persistence of various eARGs in the environment, which can be absorbed by competent cells resulting in the horizontal transfer of genes, has constantly attracted researchers’ concerns [21,26,27]. 

Human and animal guts are also major reservoirs of resistance genes. Accordingly, we suspect there would also be generous eARGs in human and animal guts, which may function effectively in the horizontal transfer of ARGs. In this study, eDNAs were successfully purified from the animal feces and contents of rabbit guts, and different classes of resistance genes and integrase genes were detected. The integrity of eDNAs is the key factor affecting their horizontal transfer. Protective adsorption by soil colloids, gravel, clay minerals, and humus would reduce the potential risk of nuclease degradation of eDNAs [37]. eDNAs in the animal intestine would be adhered to various organic materials in the guts, thus showing certain protective effects on eDNAs. Furthermore, the structural integrity of gene cassettes is directly related to their horizontal transfer and expression after the transfer and recombination. The complete sequences of genes and the compositional and structural patterns of class 1 integral cassettes were obtained by amplifying and sequencing the eDNAs isolated from the gut contents of rabbits and Wuding broiler feces in the present study. These results further indicated that the eDNAs in the animal intestine might be involved in the horizontal transfer of ARGs.

Plasmids and integrons are major vectors for the horizontal transfer of resistance genes [38–41]. Multidrug resistance genes often cluster in the form of resistance islands or gene cassettes in bacteria, and most cassettes are located on transposons or integrons in the genomes of plasmids [42–44]. These implied the selective effects of antibiotics on the mobility of resistance genes. Broad-host-range plasmids are highly abundant in animal feces such as IncP and IncQ, and plasmids IncN and IncW were repeatedly detected [45–47]. IncI1 is an important resistance gene transporter, regulating the horizontal transfer of resistance genes and pathogenic islands [48,49]. The diversity of plasmids enables the horizontal transfer of drug-resistant genes between different bacterial species. In this study, from eDNAs, plasmids such as IncP, IncN, and IncI1, etc., were also detected in animal samples, indicating that there exist extracellular plasmid genomes in animal guts involved in the horizontal transfer of resistance genes. Moreover, these plasmids are endemic plasmids that can be transmitted between distinct bacterial hosts in geographically remote countries.

Although *in vitro* transformation experiments were not done on the eDNAs extracted from the gut contents of broilers and rabbits in the present study, the larger fragments of the extracted eDNAs and amplified resistance genes, the detection of plasmid types, the sequencing results of whole reading frames of ARGs, and intact structures of integral cassettes all could implicitly indicate that eDNAs may play somewhat of a role in the horizontal transfer of resistance genes. In future research, we will conduct a series of tests on the transformation of eARGs *in vitro*.

Till now, it is unclear why the detection rates of some resistant elements in fecal eDNAs were higher than those of counterpart iDNAs, and further experiments are needed. The reason may be related to the following factors: During the experimental operation, the increased probability of exposure of fecal microbiota to the air would result in the death of strictly anaerobic bacteria and the release of DNA, thus increasing the detection rates of some resistant elements; Plasmids and other circular DNAs possess strong resistance to the harsh environment and are easy to survive, which would increase the detection rates of resistant elements carried by them; The compositions and structures of fecal flora may be varied due to animal species or individuals, so the detection rates of distinct elements would be separated. Thus, the high detection rates of eARG may indicate that the strong effect of eARGs in the pandemic of antibiotic resistance is greater than we anticipated. Our study provides new insights into the spread of drug resistance, which provides new directions for global prevention and control of the treatment ineffectiveness it brings. It should be believed that with the deepening of research, the role of eDNAs in gene horizontal transfer could ultimately be confirmed.

## Conclusion

In conclusion, the eDNAs were successfully isolated from the feces samples and intestinal contents of rabbits, and the different classes of ARGs, integrases, and plasmid replicons were detected, and the compositional and structural patterns of class 1 integral cassettes were obtained in this study. Research and elucidation of the importance of eDNAs in resistance transfer would provide a new perspective on uncovering resistance mechanisms.

## List of abbreviations

eAREs, extracellular antimicrobial resistance elements; iAREs, intracellular antimicrobial resistance elements; ARGs, antimicrobial resistance genes; iARGs, intracellular ARGs; eARGs, extracellular ARGs; HGT, horizontal gene transfer; eDNAs, extracellular DNAs; iDNAs, intracellular DNAs; iInt1, class 1 integron amplified using iDNAs; eInt1, class 1 integron amplified using eDNAs; iInt2, class 2 integron amplified using iDNAs; eInt2, class 2 integron amplified using eDNAs.
